# The impact of oncogenic mutations of the viral Src kinase on the structure and stability of the SH3 domain

**DOI:** 10.1107/S2059798321004344

**Published:** 2021-05-19

**Authors:** M. Carmen Salinas-Garcia, Marina Plaza-Garrido, Ana Camara-Artigas

**Affiliations:** a Department of Chemistry and Physics, Agrifood Campus of International Excellence (ceiA3) and CIAMBITAL, University of Almeria, Carretera de Sacramento s/n, 04120 Almeria, Spain

**Keywords:** SH3 domain, Src kinase, tyrosine kinases, intertwined dimer, oncogens, crystal structure

## Abstract

Src kinase belongs to the family of Src-related nonreceptor tyrosine kinases. Because of its physiological role in cell growth and proliferation, its activity is strictly controlled by several mechanisms. Nevertheless, in viral Src kinase (v-Src) some of these mechanisms fail, and its uncontrolled activity is responsible for the occurrence of cancer. Here, the crystal structures of three SH3-domain mutants of v-Src were determined to unveil the effects of these oncogenic mutations in this regulatory domain. Mutations in the n-Src and distal loops have a low impact on the overall structure of the domain and its capacity to form intertwined dimers. However, mutations in the RT loop compromise the stability of the domain and make the protein very prone to aggregation. Additionally, these mutations prevent the formation of intertwined dimers. The results show a synergistic effect between mutations in the RT loop and those in the n-Src and distal loops. Analysis of the structures of the v-Src SH3-domain mutants and the closed inactive conformation of cellular Src kinase (c-Src) point to a loss of the interactions that are required to establish the compact inactive form of the kinase. Nevertheless, an analysis of structures of the c-Src SH3 domain complexed with class I and II peptides points to minor changes in the interactions between the v-Src SH3 domain and these peptides. In this way, the structures reported here indicate that mutations in the RT loop might impair the kinase regulation mechanism without affecting the recognition of short proline-rich motifs in the target proteins of the kinase, thus explaining the oncogenic behaviour of the protein.

## Abstract 2

The crystal structures of three viral Src kinase (v-Src) SH3-domain variants have been determined in order to obtain insights into the roles of these oncogenic mutations in the function of v-Src.

## 1. Introduction   

Cellular Src kinase (c-Src) is one of the most representative members of the Src-family kinases (SFKs), a group of non­receptor tyrosine kinases that mediate responses to extracellular stimuli, phosphorylating a broad range of downstream substrates. These kinases are involved in several cellular processes such as proliferation, migration, differentiation and survival (Brown & Cooper, 1996 ▸; Parsons & Parsons, 2004 ▸; Bagnato *et al.*, 2020 ▸). When overactivated, they also play a role in the onset and progression of cancer. The members of this family share an overall structure organization, including a myristoylated N-terminal segment, three Src-homology (SH) domains and a C-terminal tail. The C-terminal SH1, or catalytic, domain contains an autoregulatory phosphorylation site provided by a tyrosine residue, Tyr527. When it is phosphorylated, this tyrosine binds intramolecularly to the SH2 domain. In addition, a linker region between the SH1 and SH2 domains facilitates the closed inactive conformation of the kinase by interacting with the SH3 domain. In addition, the SH3 domain recognizes short proline-rich motifs (PRMs) and regulates the activity of the enzyme, providing binding to the target proteins. The structures of several SFK members have revealed how domain interactions participate in the down-regulation of the kinases (Engen *et al.*, 2008 ▸). In this way, intramolecular interactions between the SH3, SH2 and catalytic domains facilitate the compact closed inactive conformation of the kinase.

Viral Src kinase (v-Src) was first described in chickens infected with Rous sarcoma virus (RSV). Although v-Src shares 95% sequence identity with c-Src, this kinase shows uncontrollable activity that is responsible for the occurrence of sarcoma in chickens (Levinson *et al.*, 1978 ▸). A critical difference between c-Src and v-Src is the loss of the C-terminal tail, which contains the regulatory tyrosine Tyr527 (Bjorge *et al.*, 2000 ▸). The oncogenic variant also shows several mutations in the domains of the kinase: in the myristoylated N-terminal segment (Gly62Glu and Gly63Asp), SH3 (Arg95Trp, Thr96Ile, Asp117Asn and Leu124Val) and SH1 (Arg318Gln, Thr338Ile, Ala368Asp, Val467Gly, Arg469Gly, Gln474Arg and Phe515Gln). Thus, the uncontrollable activity of v-Src is attributed to the lack of the C-terminal regulatory tail, as well as to several point mutations in its amino-acid sequence. Furthermore, biophysical characterization of v-Src demonstrated that these mutations also affect the protein stability (Falsone *et al.*, 2004 ▸).

In v-Src, the SH3 domain accumulates a significant percentage of mutations. Nevertheless, and most importantly, these mutations are critical for domain function and consequently kinase activity. Two of the mutations are located at the tip of the RT loop, which is named after the Arg95 and Thr96 residues, which are mainly conserved across the SFKs. Moreover, some residues in this loop are responsible for the specificity of the binding of PRMs and provide intramolecular contacts to facilitate the closed inactive form of the kinase. In the binding of PRMs, the canonical binding motif P*xx*P (where P is proline and *x* is any amino acid) is usually flanked on either side by a basic residue (arginine/lysine) that determines the binding orientation of the PRM by interacting with Asp99 in the RT loop (Bacarizo & Camara-Artigas, 2013 ▸). Several studies have demonstrated that when the oncogenic mutations present in the SH3 domain of v-Src are introduced individually, the variant of the kinase is inactive in cell transformation (Kato *et al.*, 1986 ▸; Miyazaki *et al.*, 1999 ▸). However, when the mutations in the RT (Arg95Trp and Thr96Ile) and n-Src (Asp117Asn) loops are combined, they make Src a highly oncogenic protein. The n-Src and RT loops enclose the hydrophobic binding surface of the SH3 domain formed by several highly conserved aromatic residues. Also, in the c-Src SH3 domain the n-Src loop acts as a hinge loop facilitating 3D domain swapping to produce intertwined dimers by interchanging the RT loop (Cámara-Artigas *et al.*, 2009 ▸). Finally, Val124 is present in the distal loop and replaces a leucine flanked by Ser123 and Thr125. The hydrogen bond between the Ser123 and Glu106 side chains plays a critical role in the folding transition state of the SH3 domain, where the folding nucleus is defined by a network of interactions between a subset of residues located in the distal loop (Klimov & Thirumalai, 2002 ▸).

In this work, we have cloned different variants of the v-Src SH3 domain in order to facilitate its structural characterization. Besides the v-Src SH3 domain bearing all of the mutations, we have cloned domains with (i) only the oncogenic mutations in the RT loop (v-Src SH3 N117D-V124L; c-Src SH3 R95W-T96I, based on the sequence of the non-oncogenic variant c-Src) and (ii) those in the n-Src and distal loop (v-Src SH3 W95R-I96T; c-Src SH3 D117N-L124V). The v-Src SH3 domain was very unstable and prone to aggregation, which impaired its purification and characterization. Previous studies showed increased stability of the c-Src SH3 domain after the introduction of a positively charged residue instead of the glutamine at position 128 (Bacarizo *et al.*, 2014 ▸). In this way, in order to stabilize the domain and to be able to crystallize the protein, we introduced a Gln128Arg mutation in the distal loop (v-Src SH3 Q128R; c-Src SH3 R95W-T96I-D117N-L124V-Q128R). Here, we discuss how the structural data can explain the behaviour of this domain and might justify the lack of control of the oncogenic v-Src tyrosine kinase.

## 2. Materials and methods   

### 2.1. Cloning, expression and purification of the v-Src SH3 domain   

**Figure 1:**
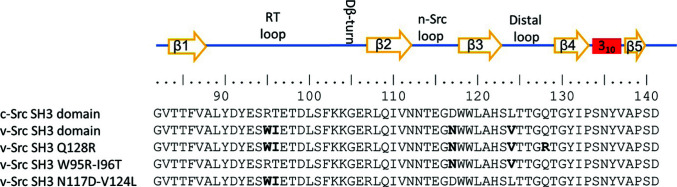
Sequences of the SH3-domain fragment of chicken c-Src and the mutants of the v-Src SH3 domain studied in this work. The v-Src residues (Schmidt Ruppin E strain; UniProt entry P00524) are shown in bold.

The genes encoding the v-Src SH3 domain and its mutants were synthesized by NzyTech, Lisbon, Portugal (Fig. 1 ▸). The synthetic genes were subcloned into the pHTP1 expression vector, including an N-terminal 6×His tag and an engineered TEV cleavage site to eliminate the histidine tag after purification (Sequeira *et al.*, 2017 ▸). The protein was expressed in *Escherichia coli* strain BL21(DE3), and purification was initially performed by a standard protocol using an Ni–NTA column (Takara Bio Europe) as described previously (Bacarizo *et al.*, 2014 ▸). However, the protein yield using the standard protocol was very low, and most of the protein remained in the cell debris. The soluble fraction of the protein was enhanced by reducing the culture temperature from 37 to 20°C before the addition of isopropyl β-d-1-thiogalacto­pyranoside. After purification with an Ni–NTA column, the protein was further purified using a Superdex 75 16/60 column equilibrated in 50 m*M* sodium phosphate, 300 m*M* sodium chloride pH 8.0 connected to an ÄKTA FPLC System (GE Healthcare Life Sciences, Barcelona, Spain). Fractions of pure protein were concentrated to 2–5 mg ml^−1^ in the same elution buffer. Before use, the protein was dialysed against the desired buffer. Although the protein yield was low, we managed to produce sufficient protein for crystallization and to solve the structures of the v-Src SH3 W95R-I96T and N117D-V124L mutants. However, the protein bearing all of the oncogenic mutations of the v-Src SH3 domain resulted in very low purification yields, and once purified the protein was very prone to forming aggregates. To improve the solubility of the protein, several constructs with fusion proteins were assayed without success. Finally, v-Src SH3 was cloned in the pHTP1 expression vector with a Q128R mutation, which resulted in a slight stabilization of the protein.

Protein purity was assessed by SDS–PAGE and the protein concentrations were established using extinction coefficients calculated using the *ProtParam* tool from ExPASy (Gasteiger *et al.*, 2005 ▸): c-Src SH3 and v-Src SH3 W95R-I96T, ɛ_280_ = 16 960 *M* ^−1^ cm^−1^; v-Src SH3, v-Src SH3 Q128R and v-Src SH3 N117D-V124L, ɛ_280_ = 22 460 *M* ^−1^ cm^−1^.

### 2.2. Crystallization and structure determination of the v-Src SH3-domain mutants   

All crystallization screens were performed with freshly purified protein to preserve the homogeneity of the samples. The v-Src SH3-domain mutants were crystallized using the vapour-diffusion technique with a sitting-drop setup. 6 µl droplets were obtained by mixing 3 µl protein solution (5–10 mg ml^−1^ in 10 m*M* Tris buffer pH 8.0) with 3 µl reservoir solution and were equilibrated against 200 µl reservoir solution. Because of the low stability of the protein, screening was performed at several different temperatures: 4, 10, 15 and 25°C. Micro-seeding techniques were used to improve the quality of the crystals, particularly for the proteins with oncogenic mutations in the RT loop. The seed stock was prepared from poor-quality crystals, which were crushed using a crystal-crusher tool (Hampton Research, USA). The crushed crystals were aspirated by pipetting and placed into a microcentrifuge tube. To ensure that the crystals were adequately crushed, a steel Seed Bead (Hampton Research, USA) was added to the microcentrifuge tube and the tube was vortexed for 1 min. The crystal solution was centrifugated at 12 000*g* for 5 min and the supernatant solution was removed. The pellet containing the crystal seeds was crushed again. Before seeding, the concentrated seed stock was diluted 100 times with 10 m*M* Tris buffer pH 8.0. Detailed descriptions of the crystallization conditions used for each protein are compiled in Table 1 ▸.

Crystals were harvested from the crystallization drop using LithoLoops (Molecular Dimensions, Sheffield, UK) and the mother liquor surrounding the crystals was carefully removed prior to flash-cooling in liquid nitrogen (Pellegrini *et al.*, 2011 ▸). Diffraction data were collected at 100 K on the BL13-XALOC beamline at the ALBA synchrotron, Barcelona, Spain (Juanhuix *et al.*, 2014 ▸) and on ID30B at ESRF, Grenoble, France (McCarthy *et al.*, 2018 ▸). Data were indexed and processed with *XDS* (Kabsch, 2010 ▸) in the *autoPROC* toolbox (Vonrhein *et al.*, 2011 ▸) and were scaled using *AIMLESS* (Evans, 2011 ▸) from the *CCP*4 suite (Winn *et al.*, 2011 ▸). Data-collection statistics are shown in Table 2 ▸.

The structures were solved using the *Phenix* suite (Adams *et al.*, 2010 ▸; Liebschner *et al.*, 2019 ▸). Molecular-replacement phasing using the *AutoMR* feature of *Phenix* (Afonine *et al.*, 2012 ▸) was performed using the coordinates of the c-Src SH3 domain in its monomeric (PDB entry 6xvn) or intertwined dimeric (PDB entry 6xvo) forms (Plaza-Garrido *et al.*, 2020 ▸). The final model was obtained after several manual building cycles in *Coot* (Emsley & Cowtan, 2004 ▸; Emsley *et al.*, 2010 ▸). Water molecules were modelled automatically using *phenix.refine* in *Phenix* (Afonine *et al.*, 2012 ▸) and manually inspected in the difference electron-density maps. In the final rounds of refinement, some molecules belonging to the precipitant solution were modelled. The final models were validated using *MolProbity* and *PDB-REDO* (Chen *et al.*, 2010 ▸; Joosten *et al.*, 2014 ▸). Structure-solution and refinement statistics are shown in Table 2 ▸. The atomic coordinates of all structures have been deposited in the Protein Data Bank (for PDB codes, see Table 2 ▸).

### 2.3. Structure analysis   

Structure superposition and r.m.s.d. calculations were performed using the *CCP*4 module *LSQKAB* (Kabsch, 1976 ▸). Protein interfaces in the crystal were characterized using the *PISA* server (Krissinel, 2011 ▸). Distances between amino acids were calculated using *CONTACT* from the *CCP*4 suite (Winn *et al.*, 2011 ▸). p*K*_a_ calculations were performed with *Rosetta* (Kilambi & Gray, 2012 ▸). Hydrogen-bond and accessible surface area (ASA) analyses were performed with the *VADAR *server (Willard *et al.*, 2003 ▸). The structural figures were generated using *PyMOL* 2.3 (Schrödinger).

### 2.4. Fluorescence measurements   

#### 2.4.1. Acid and alkaline denaturation of the protein   

All fluorescence spectra were collected using a Perkin Elmer LS-50 spectrofluorimeter. The stability of the protein versus pH was assessed by measuring the intrinsic fluorescence of the protein. The protein concentration was 2 µ*M* in 50 m*M* buffer prepared using the corresponding salts and acids: pH 2.0–3.0, phosphoric acid; pH 3.0–4.0, formic acid; pH 4.0–5.5, acetic acid; pH 6.0–7.0, NaH_2_PO_4_; pH 7.5–9.0, Tris acid; pH 9.5–11.0, Na_2_CO_3_; pH 11.5–13.0, Na_3_PO_4_. Appropriate blank corrections were made in all spectra. At least two independent measurements were conducted at each pH value, and the actual pH value of each sample was measured with a pH meter after completion of the experiment. Samples were excited at 280 nm, and the emission spectra were collected between 300 and 500 nm. The bandwidth for slits was 5 nm for both excitation and emission, and the path length was 1 cm. The protein stability versus pH was analysed at 25°C in the pH range 1–14, as reported previously (Plaza-Garrido *et al.*, 2020 ▸). The apparent p*K*_a_ value of the acid and basic transition can be calculated using the Henderson–Hasselbalch equation,
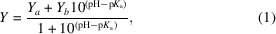
where *Y* is the fluorescence intensity observed and *Y*_*a*_ and *Y*_*b*_ are the fluorescence intensity at the lowest and highest pH value, respectively. The experimental values were fitted to (1) using *Origin* 2018 (OriginLab, USA).

#### 2.4.2. Guanidine hydrochloride denaturation   

The chemical-induced unfolding of the v-Src SH3-domain variants was measured in the presence of the denaturant guanidine hydrochloride (GndHCl). Briefly, 2 µ*M* protein samples were prepared in 50 m*M* sodium phosphate pH 7.0 containing different concentrations of GndHCl and left overnight at 25°C to reach equilibrium. At least two independent measurements were conducted at each GndHCl concentration. The unfolding curves were analysed using the two-state model (Pace & Laurents, 1989 ▸). Thus, the value of the Gibbs energy in the absence of denaturant, Δ*G*_w_, was obtained using the equation
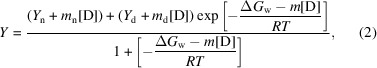
where *Y* is the spectral energy of emission, [D] is the de­naturant concentration and *m* is the slope. *Y*_n_ and *Y*_d_ are the spectral energy emission values of the native and unfolded protein, respectively. The experimental values were fitted to (2) using *Origin* 2018 (OriginLab, USA).

### 2.5. Amyloid characterization   

To characterize the presence of amyloid aggregates in the purified protein, we used the thioflavin T (ThT) enhancement of fluorescence (Hatters & Griffin, 2011 ▸). A stock solution of 2 m*M* ThT in water was prepared by weighing. The dye concentration was experimentally determined by measuring the absorbance at a wavelength of 412 nm and using a molar extinction coefficient of 36 000 *M* ^−1^ cm^−1^. Fluorescence spectra were collected on a Perkin Elmer LS-50 spectrofluorimeter using a 3 mm path-length Hellma quartz-cell microcuvette. For each measurement, 3–5 µl protein solution (final concentration 15 µ*M*) was mixed with 0.5 µl ThT stock solution in a final volume of 100 µl buffer (100 m*M* sodium chloride, 50 m*M* sodium phosphate pH 7.0). Fluorescence emission spectra were acquired in the range 450–600 nm with a 10 nm bandwidth upon excitation at 445 nm using a 5 nm bandwidth.

We also determined the presence of amyloids by measuring the change in the absorption spectrum of the dye Congo Red (CR) in the visible range (Hatters & Griffin, 2011 ▸). The CR spectra were recorded between 400 and 600 nm using a Perkin Elmer Lambda 25 spectrometer. A stock solution of 100 µ*M* CR was prepared in double-distilled water. For each measurement, a volume of between 50 and 100 µl protein solution (final concentration 50 µ*M*) was mixed with 100 µl CR stock solution and adjusted to a final volume of 500 µl with 50 m*M* sodium phosphate pH 7.0.

### 2.6. Dynamic light scattering (DLS)   

DLS experiments were performed in a Zetasizer Nano instrument (Malvern Instruments, United Kingdom) equipped with a 10 mW helium–neon laser (wavelength 632.8 nm) and a thermoelectric temperature controller. Experiments were analysed with the *Zetasizer* software (Malvern Instruments). The hydrodynamic radius (*R*_h_) was determined at two different protein concentrations (5 and 10 mg ml^−1^) as described elsewhere (Bacarizo *et al.*, 2014 ▸). Experiments were conducted at three different temperatures (15, 20 and 25°C) and two pH values (pH 5.0 in 50 m*M* acetate buffer and pH 7.0 in 50 m*M* phosphate buffer).

## 3. Results   

### 3.1. Structure of the v-Src SH3-domain mutants   

**Figure 2:**
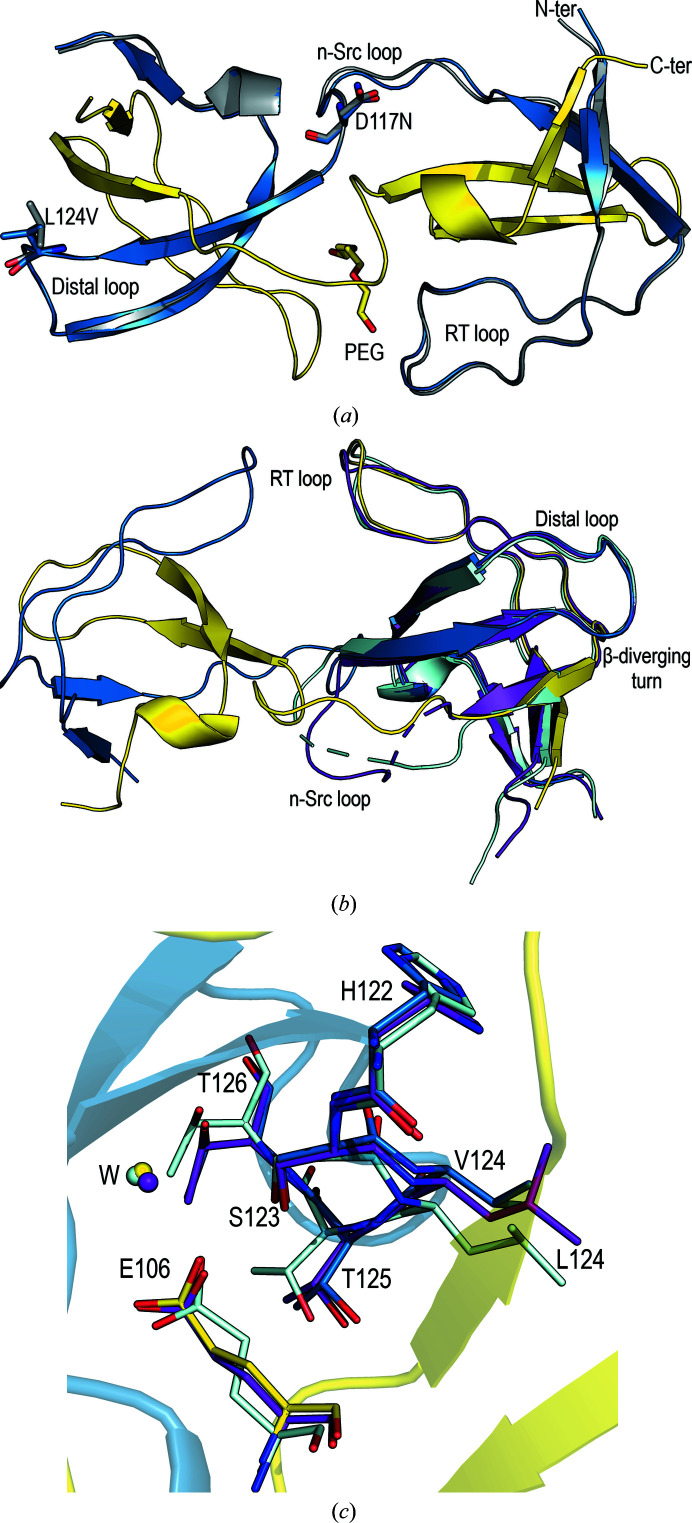
(*a*) Intertwined dimer of the v-Src SH3 W95R-I96T variant (PDB entry 7net; chain *A*, blue; chain *B*, yellow) overlaid on the c-Src SH3-domain open protomer (chain *A*, grey; PDB entry 6xvo). The average backbone r.m.s.d. value is 0.28 Å. Mutated residues in the n-Src and distal loops are shown as sticks (chain *A*). (*b*) Superposition of the v-Src SH3 W95R-I96T variant intertwined dimer (chain *A*, blue; chain *B*, yellow) and the c-Src SH3-domain monomer (chain *A*, purple; chain *B*, cyan; PDB entry 6xvo). (*c*) Residues in the distal loop are shown as sticks.

The crystals of v-Src SH3 W95R-I96T (c-Src SH3 D117N-L124V) belonged to space group *P*6_5_, with unit-cell parameters similar to those obtained for crystals of the c-Src SH3-domain intertwined dimers (Bacarizo *et al.*, 2014 ▸; Cámara-Artigas *et al.*, 2009 ▸). The coordinates of the open protomer of c-Src were used for molecular replacement (PDB entry 6xvo), and the search resulted in the placement of two chains of the open protomer in the asymmetric unit. Comparison of this dimer with those previously reported did not show significant differences. Both mutations, Asp117Asn and Leu124Val, do not produce significant changes in the backbone of the protein (Fig. 2 ▸). As in the previous structures, a low-molecular-weight PEG is positioned in the dimer interface, where a cluster of interactions stabilize the domain-swapped form of the protein (Cámara-Artigas *et al.*, 2009 ▸). Among the most relevant interactions are an inter-chain salt bridge between Arg95 and Glu115 and a cluster of hydrogen bonds where Thr96 partici­pates in intra-chain and inter-chain interactions.

A comparison of the v-Src SH3 W95R-I96T dimer structure with the monomeric structure of the c-Src SH3 domain shows a different scenario. In the distal loop, Leu124 is placed between Ser123 and Thr125-Thr126. These residues form a cluster of hydrogen bonds with Glu106 at the β-diverging turn that plays a critical role in folding the c-Src SH3 domain (Grantcharova *et al.*, 1998 ▸). The structures of the monomeric form of the c-Src SH3 domain were obtained in the monoclinic space group *P*2_1_ with two (PDB entry 6xvn) or four (PDB entry 6xvm) molecules in the asymmetric unit (Plaza-Garrido *et al.*, 2020 ▸). These chains show the characteristic SH3 fold with different conformations in the distal loop, where the main difference is the hydrogen-bond network involving residues Glu106, Ser123 and the neighbouring residues Thr125 and Thr126 (Bacarizo *et al.*, 2014 ▸). In these conformations, the Leu124 side chain adopts different conformers, facilitating the rearrangement of hydrogen bonds. The replacement of leucine by valine, which has a shorter chain, might impair the ability of this residue to act as a switch between conformations (Fig. 2 ▸).

**Figure 3:**
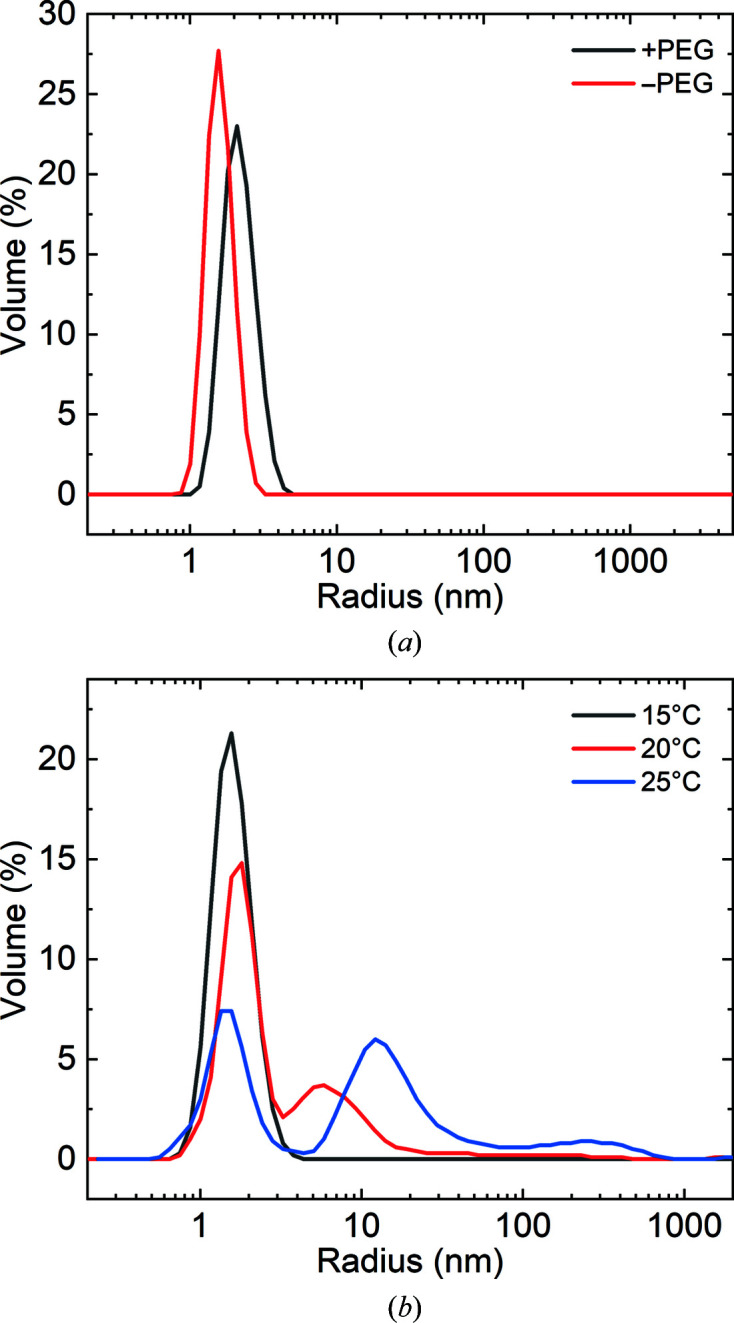
DLS measurements of the v-Src SH3 variants. (*a*) v-Src SH3 W95R-I96T at 5 mg ml^−1^ in 50 m*M* sodium acetate buffer pH 5.0 in the absence (red line) and in the presence (black line) of 5% PEG 300 at 25°C. In the absence of PEG, the protein is a monomer with *R*_h_ = 1.8 ± 0.3 nm. After adding 5% PEG 300, the hydrodynamic radius increases to a value of 2.4 ± 0.5 nm. (*b*) Temperature-dependent aggregation of the v-Src SH3 N117D-V124L variant. After incubation for 24 h at three different temperatures, the protein was measured at 5 mg ml^−1^ in 50 m*M* sodium phosphate buffer pH 7.0. At low temperature (≤15°C) 99.9% of the protein is a monomer with an *R*_h_ of 1.8 ± 0.4 nm. At higher temperatures, the protein aggregates, forming high-molecular-weight oligomers, and after one day of incubation the monomer population decreases at both 20°C (66%) and 25°C (39%). These oligomers tested positive for amyloid aggregates using the ThT and CR assays.

Crystals of the v-Src SH3 domain bearing oncogenic mutations in the RT loop, the v-Src SH3 N117D-V124L (c-Src SH3 R95W-T96I) and Q128R (c-Src SH3 R95R-T96I-D117N-L124V-Q128R) variants, are more challenging to crystallize because these proteins are very prone to form high-molecular-weight aggregates. v-Src SH3 W95R-I96T shows a single population (99.9%) with a hydrodynamic radius characteristic of a monomer (*R*_h_ = 1.8 nm) and forms dimers in the presence of PEG 300 (*R*_h_ = 2.4 nm; Fig. 3 ▸ *a*). However, the protein with mutations in the RT loop shows a temperature-dependent aggregation process, giving a single peak with an *R*_h_ of 1.8 nm when kept at temperatures lower than 15°C. Upon exposure to 20 and 25°C for one day, the DLS measurements show a decrease in the peak corresponding to the monomer and an increase in the aggregate peaks (Fig. 3 ▸ *b*). ThT and CR assays showed that both proteins form amyloids in a few days at 25°C under mildly acidic and neutral pH conditions. For this reason, all crystallization trials were performed between 4 and 15°C.

**Figure 4:**
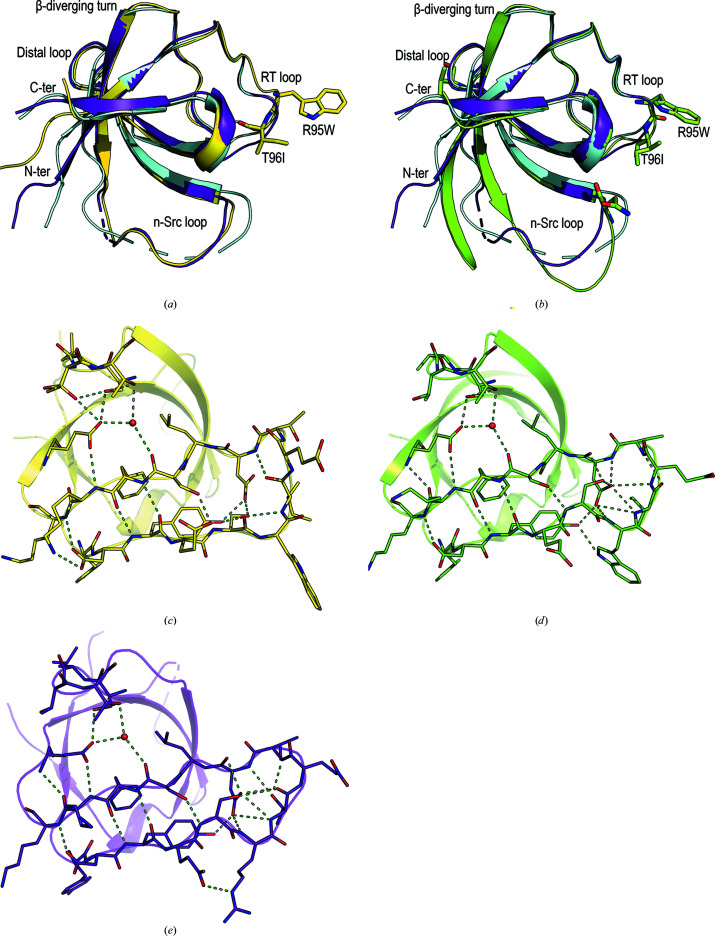
Cartoon representation of the superposition of the c-Src SH3 domain (PDB entry 6xvn; chain *A*, purple; chain *B*, cyan) and (*a*) the v-Src SH3 N117D-V124L variant (PDB entry 7nes) and (*b*) the v-Src SH3 Q128R variant (PDB entry 7ner). In both chains of the c-Src SH3 domain, the n-­Src loop has been partially modelled and the unmodelled residues are represented by a dashed line. Hydrogen bonds in the RT loop of (*c*) the v-­Src SH3 N117D-V124L variant, (*d*) the v-Src SH3 Q128R variant and (*e*) the c-Src SH3 domain (chain *A*).

The v-Src SH3 N117D-V124L crystals belonged to the trigonal space group *P*3_2_21, with only one SH3 molecule in the asymmetric unit. The overall fold corresponds to the closed-monomer form of the SH3 domain. Superposition of the v-Src SH3 N117D-V124L chain on both chains present in the monomeric form of c-Src SH3 (PDB entry 6xvn) shows backbone r.m.s.d. values of 0.84 and 1.01 Å for chains *A* and *B*, respectively. In this mutant, the n-Src loop conformation is similar to that found in chain *A* of c-Src SH3. Meanwhile, the distal loop conformation differs from that present in both chains of c-Src SH3 (Fig. 4 ▸). As expected, the most significant differences are found in the RT loop, in which the oncogenic mutations Arg95Trp and Thr96Ile are located. In c-Src SH3, the hydrogen bond in the type I β-turn occurs between Thr96 and Asp99 through their side-chain and backbone atoms, respectively. Nevertheless, in v-Src SH3 the substitution of threonine by isoleucine prevents hydrogen-bond formation by its side chain, and in this case the type I β-turn shows a hydrogen bond between the backbone atoms Ile96 O and Asp99 N (Fig. 4 ▸).

Although we tried to purify the protein bearing all of the oncogenic mutations, v-Src SH3, this protein was very prone to aggregation, impairing its purification and crystallization. To analyse the effect of the oncogenic mutations on the overall structure of this domain, we introduced a Gln128Arg mutation in the distal loop, which increases the stability of c-Src SH3 (Bacarizo *et al.*, 2014 ▸). The v-Src SH3 Q128R mutant was stable enough to allow biophysical studies and to be crystallized. Even with this stabilizing mutation, the protein was still very prone to aggregation, forming amyloid-like aggregates. All of the crystallization trials were performed at 4 and 10°C using fresh protein. The initial crystals were twinned and the crystal quality was improved by micro-seeding (Bergfors, 2003 ▸). The resulting crystals belonged to the monoclinic space group *P*2_1_, with only one SH3 molecule of the monomer form in the asymmetric unit. Comparison of the backbone atoms of this structure with those in the monomeric structure of c-Src SH3 (PDB entry 6xvn) shows average r.m.s.d. values of 0.68 and 1.11 Å compared with chains *A* and *B*, respectively. Significant differences are found in conformations of the RT, n-Src and distal loops. The type of β-turn in the RT loop is also different from that found in v-Src SH3 N117D-V124L. In this case, residues Ile96–Asp99 form a type IV β-turn with a hydrogen bond between Ile96 N and the Asp99 side chain (Fig. 4 ▸). It is worth mentioning that in this structure Trp95 and Glu97 are in unfavourable, but allowed, regions of the Ramachandran plot.

Besides the RT loop mutations, the v-Src SH3 Q128R variant also bears oncogenic mutations in the n-Src and distal loops at Asn117 and Val124. The conformation of its n-Src loop differs from those found in c-Src SH3 and v-Src SH3 N117D-V124L. Moreover, in the n-Src loop all of the residues have been modelled. Several hydrogen bonds are responsible for this reduced flexibility. Asn117 interacts with Ser134 in the 3_10_-helix, and a hydrogen bond between Asn112 and Gly81 also reduces the flexibility of the amino-terminal region. In c-Src SH3 the amino-terminal region is usually disordered, but in v-Src SH3 Q128R residues Gly81–Leu89 form an extended β1 strand. Comparison of the v-Src SH3 Q128R and v-Src SH3 N117D-V124L variants also shows differences in the hydrogen-bond network around Glu106. In the Q128R mutant, Thr125 shows a single conformation and does not form a hydrogen bond to the Glu106 side chain (Fig. 4 ▸).

Finally, analysis of the crystal interfaces of the monomeric and dimeric structures of the v-Src SH3 variants using the *PISA* server shows no crystallographic correlation between these crystal structures. The intertwined dimer structure crystallizes in the same space group as other c-Src SH3 intertwined dimers described previously and the crystallo­graphic interfaces are practically the same (Cámara-Artigas *et al.*, 2009 ▸). However, the monomeric structures of the v-Src variants belong to crystals that differ from those used to obtain previous structures of the c-Src SH3 domain. Moreover, both crystals show an unusually low solvent content (v-Src SH3 Q128R and v-Src SH3 N117D-V124L have 29% and 35% solvent content, respectively). v-Src SH3 Q128R has up to six crystallographic interfaces, while v-Src SH3 N117D-V124L has only three. In addition to the low solvent content, in v-Src SH3 Q128R a PEG molecule and a sulfate ion facilitate the packing between protein molecules in the crystal. In both crystal forms, Trp95 and Ile96 participate in the crystallo­graphic interfaces, but the packing around these residues is different in each crystal form, which results in different rotamers of their side chains and displacement of the backbone atoms (the r.m.s.d. of the backbone atoms is ∼2 Å). Some residues in the n-Src loop also participate in the crystallo­graphic interfaces, and the tight packing in the crystal reduces the flexibility of this loop, which has been fully modelled. The conformation of this loop is different in each monomeric structure, which is also related to the contacts between secondary-structure elements in the v-Src SH3 Q128R variant, as explained above.

### 3.2. Biophysical characterization of the v-Src mutants   

#### 3.2.1. Stability of the v-Src SH3 mutants versus pH   

**Figure 5:**
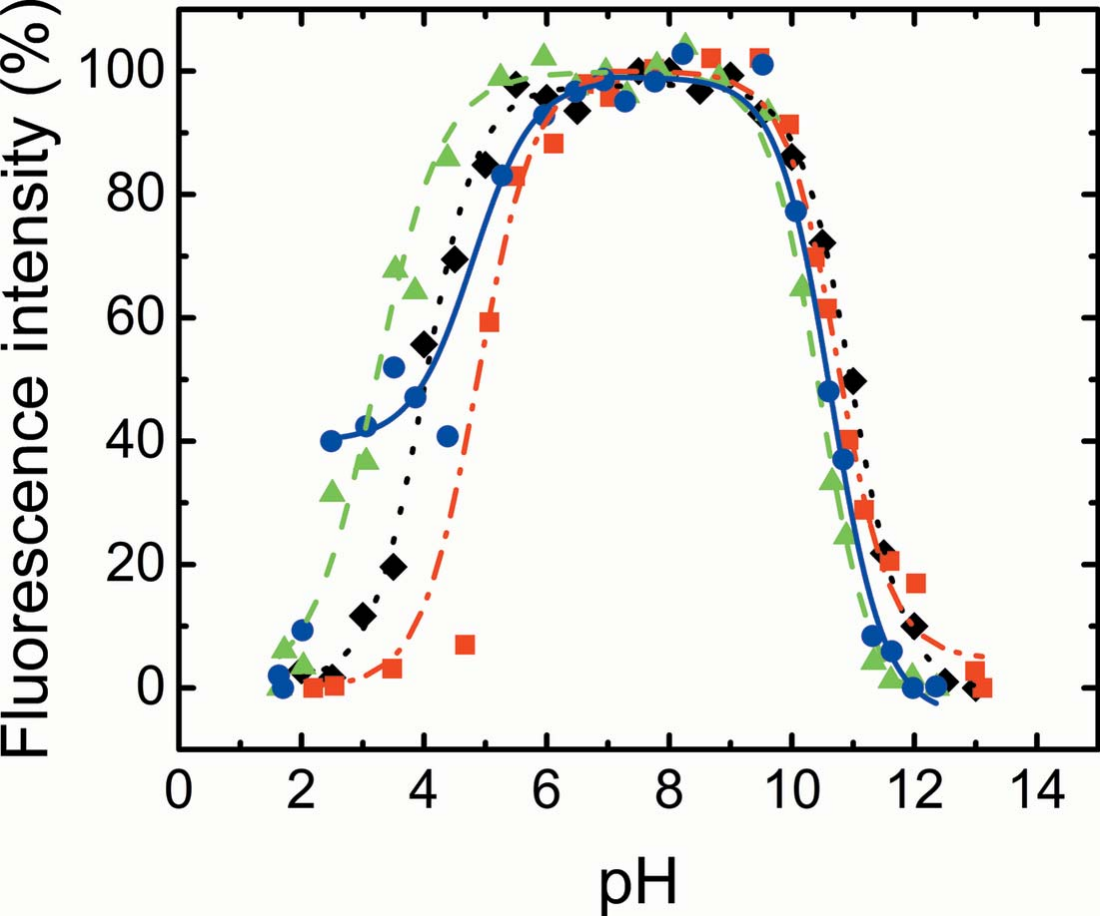
Native fraction of the v-Src SH3 mutants versus pH at 25°C: v-Src SH3 Q128R (blue circles and line), v-Src SH3 W95R-I96T (green triangles and dashed line) and v-­Src SH3 N117D-V124L (red squares and dashed/dotted line). For comparison, data for c-Src SH3 have also been included (black diamonds and dotted line).

We determined the stability of the v-Src SH3 variants versus pH by measuring the intrinsic fluorescence spectra in the pH range 1–14. Fig. 5 ▸ shows the normalized fluorescence emission intensity versus pH, and Table 3 ▸ compiles the apparent p*K*_a_ values obtained for the acidic and basic denaturation of the proteins as calculated using (1).

The pH-dependence of stability of the v-Src SH3 variants shows the typical bell-shaped curve. In the acidic region, the transition might be attributed to the protonation of aspartate and glutamate residues. The v-Src SH3 W95R-I96T mutant is the most stable compared with the proteins bearing the oncogenic mutations in the RT loop. In addition, in the n-Src loop the presence of asparagine instead of aspartate seems to produce some stabilization in this mutant compared with c-Src SH3. Asn117 forms hydrogen bonds to Ser134 in the 3_10_-helix which are not dependent on the pH, and might confer higher stability on this mutant in the acidic region. However, the v-Src SH3 Q128R and N117D-V124L mutants are prone to aggregate in acidic solutions and have a narrower stability range (pH 5.5–10). In the basic range all of the v-Src SH3 variants show the same behaviour, with an apparent p*K*_a_ of ∼10.6 ± 0.2, which is similar to that obtained with the non-oncogenic protein c-Src SH3 (Plaza-Garrido *et al.*, 2020 ▸).

We calculated the apparent p*K*_a_ values of ionizable residues using the *Rosetta* server (Kilambi & Gray, 2012 ▸; Supplementary Table S1). In all of the v-Src SH3 structures Asp99 shows a p*K*_a_ value lower than its intrinsic p*K*_a_ (4.0), and it is the residue with the most remarkable changes in all of the analysed structures. Asp99 plays a critical role in binding PRMs by forming a salt bridge with the positively charged residue flanking the canonical sequence P*xx*P (Bacarizo & Camara-Artigas, 2013 ▸). The RT loop must have some flexibility to perform its function in recognizing PRMs, and the interactions between residues in this loop determine its final conformation. In this way, Asp99 can establish up to five hydrogen bonds through its carboxylate side chain, and the low ASA value of its side chain (<10 Å^2^) indicates complete burial of the residue (Fig. 4 ▸). An explanation of the low p*K*_a_ value calculated for Asp99 is that some conformations of the RT loop might result in a tightly packed core that fits the deprotonated residue better (Kilambi & Gray, 2012 ▸). Upon the binding of PRMs, the network of hydrogen-bond interactions around Asp99 is modified to facilitate the salt bridge between Asp99 and the flanking arginine residue in the PRM (Bacarizo *et al.*, 2015 ▸). Interestingly, although Asp99 forms a salt-bridge interaction in the complex structures of c-Src SH3 and PRMs, this residue does not form interdomain inter­actions in the structures of the closed inactive conformation of the full kinase (Williams *et al.*, 1997 ▸; Sicheri *et al.*, 1997 ▸).

#### 3.2.2. Isothermal denaturation by guanidium hydrochloride   

**Figure 6:**
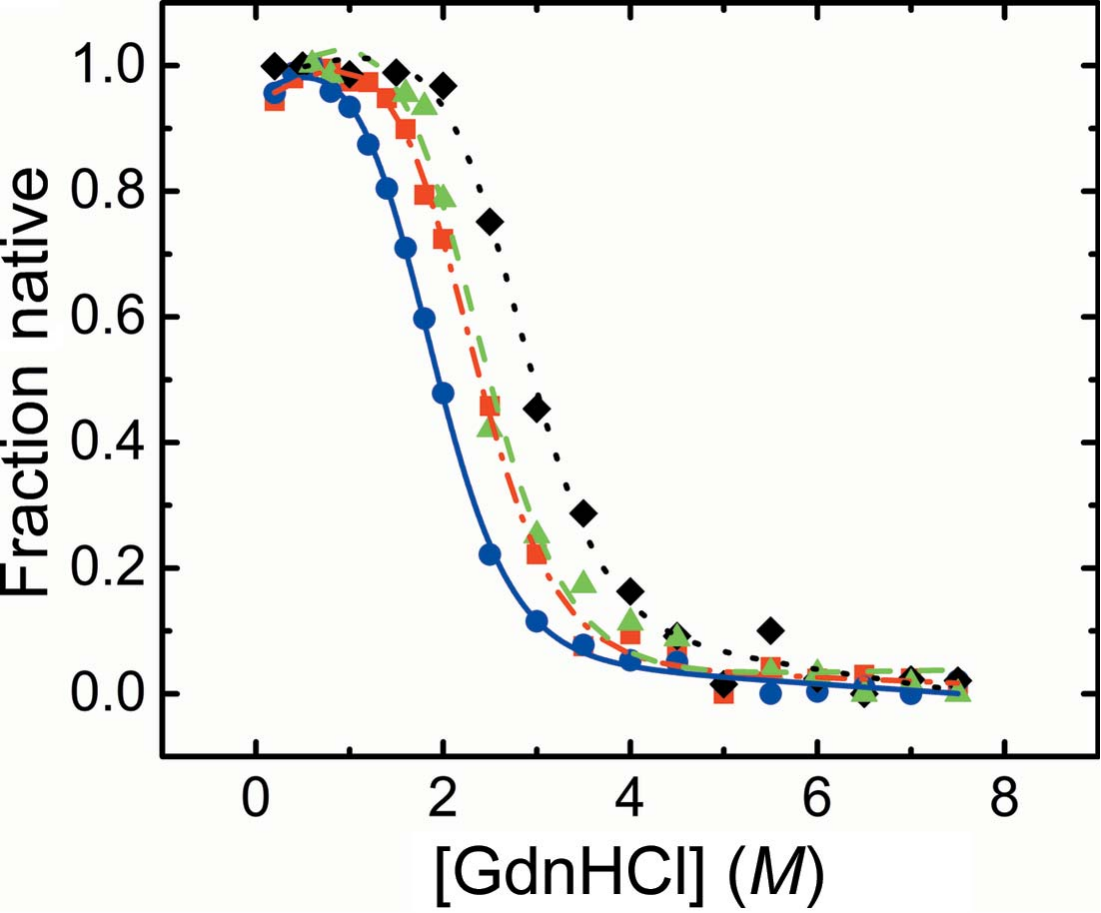
Native fraction of v-Src SH3 mutants versus GndHCl concentration at pH 7.0 and 25°C: v-Src SH3 Q128R (blue circles and line), v-Src SH3 W95R-I96T (green triangles and dashed line) and v-Src SH3 N117D-V124L (red squares and dashed/dotted line). For comparison, the unfolding curve of c-Src SH3 (black diamonds and dotted line) has also been included.

The GdnHCl-induced equilibrium denaturation of v-Src SH3 mutants was monitored by the decrease in the intrinsic fluorescence of the protein at 25°C (Fig. 6 ▸). The unfolding curves were analysed using the two-state model, and the thermodynamic parameters are compiled in Table 4 ▸. The midpoints of the transition of the N117D-V124L and W95R-I96T variants and their unfolding free-energy change are practically the same but are lower than the values for c-Src SH3 (Plaza-Garrido *et al.*, 2020 ▸). The protein with all of the oncogenic mutations, v-Src SH3 Q128R, shows the lowest stability.

## 4. Discussion   

### 4.1. The structures of the v-Src SH3-domain variants   

Src kinases play an important role in the cell, mediating responses to extracellular stimuli by phosphorylating a broad range of downstream substrates, and are strictly regulated by several mechanisms (Roskoski, 2004 ▸). In the v-Src kinase, a few mutations impair these regulatory mechanisms, resulting in an oncogenic protein. A number of these mutations are located in the SH3 domain, which plays a critical role in enzyme function by facilitating the interaction with partner proteins and providing the formation of the closed inactive conformation of the kinase. It has been reported that the individual introduction of the oncogenic mutations Arg95Trp, Thr96Ile, Asp117Asn and Leu124Val does not modify the cellular function of c-Src. However, when these mutations were combined, the kinase was active and highly oncogenic (Miyazaki *et al.*, 1999 ▸). To study the structural changes produced by the presence of these oncogenic mutations in the SH3 domain of the c-Src tyrosine kinase, we have crystallized and solved the structures of several variants of the v-Src SH3 domain. The protein bearing all of the oncogenic mutations showed very low stability, which precluded its purification and therefore its crystallization. To increase the stability of the protein, we used a v-Src SH3 mutant in which glutamine was replaced by arginine at position 128. We have previously demonstrated that this substitution increases the stability of c-Src SH3 without affecting the formation of domain-swapped dimers (Plaza-Garrido *et al.*, 2020 ▸; Bacarizo *et al.*, 2014 ▸).

**Figure 7:**
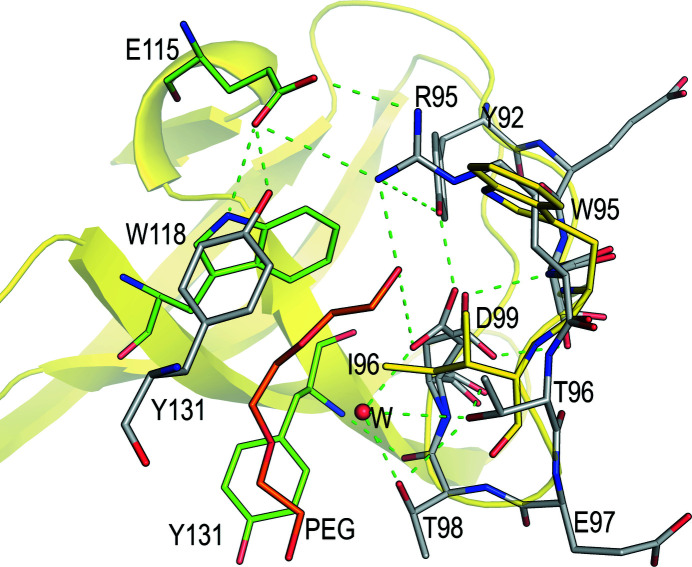
Interactions at the interface of the intertwined dimer of v-Src SH3 W95R-I96T (PDB entry 7net). The residues in the different chains of the dimer are coloured grey (chain *A*) and green (chain *B*). The structure of the v-­Src SH3 Q128R mutant (yellow; PDB entry 7ner) is overlaid on one of the dimer chains. The mutated residues, Trp95 and Ile96, are shown as sticks. The Thr96(*A*)/Thr98(*A*) residues form a hydrogen bond mediated by a water molecule (W) to the symmetry-related Thr96(*B*)/Thr98(*B*) residues. For the sake of clarity, these symmetry-related residues are not represented. Asp99 show a double conformation, which facilitates intra-chain hydrogen bonds with different residues in the RT loop.

We obtained an intertwined structure of the v-Src SH3 W95R-I96T variant, which shows the same overall fold as previous domain-swapped dimers of c-Src SH3, and the mutated residues do not produce significant changes in the structure (Cámara-Artigas *et al.*, 2009 ▸; Bacarizo *et al.*, 2014 ▸; Plaza-Garrido *et al.*, 2020 ▸). However, we could not obtain the intertwined dimer structure of v-Src SH3 bearing the oncogenic mutations in the RT loop, even with crystals obtained using the same crystallization conditions. Instead, we only obtained the monomeric form, and both mutants had only a single molecule in the asymmetric unit. The reason for the lack of intertwined dimers of these mutants can be found in the role played by Arg95 and Thr96 in the stabilization of the dimeric structure. Neither tryptophan nor isoleucine can form interactions to stabilize the intertwined dimer (Fig. 7 ▸).

It is worth mentioning that the monomeric form of c-Src SH3 crystallizes with two or four molecules in the asymmetric unit that represent two different main conformations of the domain (Plaza-Garrido *et al.*, 2020 ▸). In the structures of c-Src SH3, the n-Src loop shows high flexibility that impairs the modelling of the whole backbone/side chains of the residues in the loop, but this flexibility is lost in the v-Src SH3 Q128R mutant. In the RT loop, the oncogenic mutations modify the network of interactions around Asp99, and this results in a different loop conformation compared with the c-Src structures. Arg95 and Thr96 are conserved in the SFKs, except for the Hck kinase, in which isoleucine and histidine instead of arginine and threonine result in a noticeable increase in the flexibility of the RT loop (Arold *et al.*, 1998 ▸). In the v-Src SH3 Q128R structure, the loss of intra-chain interactions in the RT loop might increase the energy of the loop. Besides, the stability of this mutant might be affected by the presence of Trp95 and Glu97 in unfavourable regions of the Ramachandran plot. Tryptophan is not a typical residue in β-turns, where proline and glycine residues are statistically favoured, presumably because their unique side chains contribute favourably to the conformational stability of the β-turn. Additionally, if the loop is solvent-exposed then polar residues are preferred to stabilize the β-turn (Trevino *et al.*, 2007 ▸). An example of a tryptophan in a solvent-exposed β-turn is Trp60 in the highly amyloidogenic protein β2-microglobulin. The Trp60Gly mutation stabilizes the protein and reduces its amyloidogenic propensity (Esposito *et al.*, 2008 ▸). In our case, the oncogenic mutation Arg95Trp might have the opposite effect, reducing the stability of the protein and increasing its amyloidogenic propensity.

Folding experiments conducted with c-Src mutants with a cross-linked RT loop and N- and C-termini showed a dramatic slowdown in the unfolding rate, suggesting that the rate-limiting step in unfolding involves dissociation of the N- and C-termini and opening of the RT loop (Grantcharova *et al.*, 2000 ▸). Experimental and molecular-dynamics simulation studies showed that the first regions of the SH3 domain to become ordered are the three hairpin loops: the distal loop, RT loop and n-Src loop (Baker *et al.*, 1999 ▸). The three-stranded sheet formed by the distal loop β-hairpin and the n-Src loop contains the residues that are considered to be in the hydrophobic folding nucleus of the SH3 domain (Ile110, Ala121 and Ile132). This sheet has a much higher density of stabilizing interactions than other portions of the protein with similar length, and the ordering of the residues produces significant gains in attractive interactions. Meanwhile, the ordering of additional residues in the RT loop increases the entropic cost of structure formation without significant increases in the attractive native interactions (Northey *et al.*, 2002 ▸; Riddle *et al.*, 1999 ▸). The interaction of the RT loop with the central three-stranded sheet is facilitated by hydrophobic contacts between the core residues, Phe102 and Leu108, and a hydrogen bond between the side chain of Glu106 and the backbone N atom of Lys103. The weak interactions between the folding nucleus and the late folding of the RT loop might explain the easy interchange of this loop in forming the intertwined dimers.

In the monomeric structures of v-Src SH3, the RT loop conformation differs from that found in the c-Src SH3 domain. The disruption of some interactions in the RT loop might also affect the stability of the protein. The electrostatic inter­actions in the RT loop are also altered by the presence of two hydrophobic residues, tryptophan and isoleucine, instead of the polar residues arginine and threonine. The p*K*_a_ value of Asp99 might be affected by these changes and it might explain the shorter range of stability versus pH of v-Src mutants bearing the oncogenic mutations in the RT loop.

### 4.2. SH3 interactions in the kinase and in complex with PRMs   

**Figure 8:**
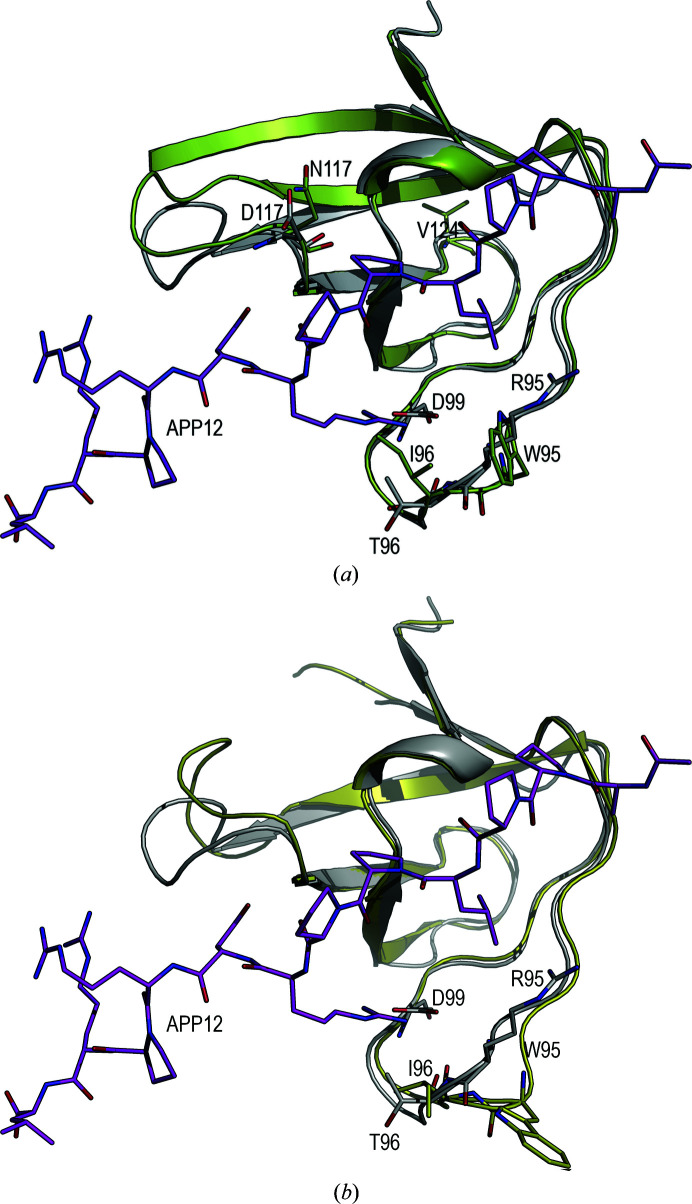
Superposition of the (*a*) v-Src SH3 Q128R (green) and (*b*) v-Src SH3 N117D-V124L (yellow) variants with the structure of the c-Src SH3 Q128R–APP12 complex (PDB entry 5ob1). The SH3 domain is represented as a cartoon (white) and the peptide as sticks (magenta). The mutations present in each oncogenic variant are represented as sticks. The Asp99–APP12–Arg7 salt bridge that drives the peptide orientation upon binding is also shown.

**Figure 9:**
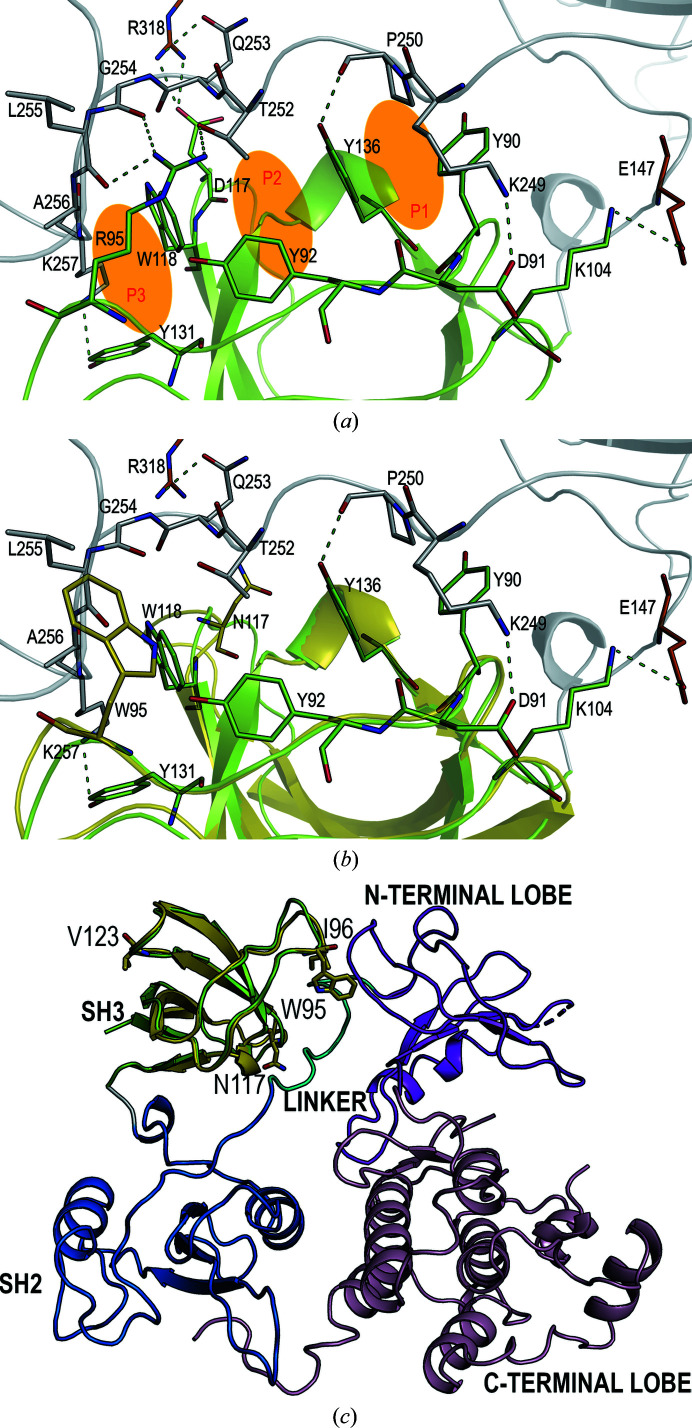
(*a*) Interactions between the SH2–kinase linker (grey) and the SH3 domain (green). Residues of the SH2 and kinase domains that also interact with the SH3 domain in the structure of the c-Src kinase (PDB entry 2ptk) are shown as orange sticks. The interactions between residues in the SH3 domain and SH2–kinase linker (shown as sticks) are marked by green dashed lines. The three interaction pockets are marked in orange and labelled P1, P2 and P3. (*b*) Superposition of the SH3 domain of the c-Src kinase and the v-Src SH3 Q128R mutant (PDB entry 7ner, yellow, cartoon). The oncogenic mutations Trp95 and Asn117 are shown as yellow sticks and the interactions established by Arg95 and Asp117 have been removed. (*c*) Closed inactive conformation of the c-Src tyrosine kinase from chicken (PDB entry 2ptk). The SH1-domain N- and C-­terminal lobes are coloured magenta and pink, respectively. The SH2 domain is coloured blue and the SH2–SH1 linker cyan. The SH3 domain is shown in green and the superposition of v-Src SH3 is shown in yellow. The mutated residues in the SH3 domain are shown as sticks.

Previously, it has been shown that the oncogenic mutations in the SH3 domain suppress binding to the c-Src kinase domain while retaining the ability to bind a subset of cellular proteins at a level similar to that of c-Src SH3 (Miyazaki *et al.*, 1999 ▸). Residues in the RT loop are responsible for the specificity of the binding, and a salt bridge between Asp99 and an arginine flanking the canonical P*xx*P motif determines the orientation of the peptide as class I or II (Fig. 8 ▸; Bacarizo & Camara-Artigas, 2013 ▸). However, Asp99 does not interact with the linker region of the c-Src kinase (PDB entry 2ptk; Williams *et al.*, 1997 ▸). Indeed, this region only has a proline residue, Pro250, which together with the aliphatic chain of Lys249 interacts with the first pocket (Tyr90/Tyr136) in the hydrophobic surface of the SH3 domain. The methyl group of Thr252 occupies the second binding pocket (Tyr136/Pro133/Trp118) and Ala256-Lys257 make a stacking inter­action with the third pocket (Trp118/Tyr131) (Fig. 9 ▸ *a*). In this way, the interaction of the SH2–kinase linker is expected to be weaker than the binding of the PRMs of the partner proteins with the consensus sequence P*xx*P. The inactive closed state of the kinase shows two salt bridges that can compensate for the lack of the second proline residue to drive intramolecular binding (Asp91–Lys249 and Asp117–Arg318). The oncogenic mutation Asp117Asn impairs the salt bridge with Arg318; also, the replacement of arginine by tryptophan, Arg95Trp, results in the loss of several interactions between the linker region and the RT loop (Fig. 9 ▸ *b*). Therefore, these interactions might be essential to support the weak contact between the SH3 domain and the linker region and would explain the previous results that point to a lack of binding of the v-Src SH3 domain to the linker region in the v-Src kinase (Fig. 9 ▸ *c*; Miyazaki *et al.*, 1999 ▸). However, residues Arg95-Thr96 do not interact with residues in the PRMs in the complex structures of class I and II peptides with c-Src SH3 (Bacarizo & Camara-Artigas, 2013 ▸; Fig. 8 ▸). In this way, the interaction of the v-Src SH3 domain with its partner proteins might not be affected, as observed previously (Miyazaki *et al.*, 1999 ▸).

Although 3D domain-swapping has been described in the c-Src SH3 domain, to date there is no evidence of the formation of intertwined structures in the full Src kinase. Other SH3 domains from proteins with different structures and functions also form intertwined dimers (Cámara-Artigas, 2016 ▸; Richter *et al.*, 2020 ▸). Moreover, this oligomerization process has also been described in the SH2 domain (Huculeci *et al.*, 2015 ▸). Given the repeated occurrence of this oligomeric association in these domains, the question arises of whether there might be an associated biological function and how this oligomerization would affect the kinase function. Comparative studies performed with c-Src and v-Src demonstrated that the viral form has a larger number of unstructured regions, a lower compactness, a higher exposure of hydrophobic residues, an increased sensitivity to denaturation and a more pronounced tendency towards aggregation (Falsone *et al.*, 2004 ▸). In c-Src, the formation of the intertwined oligomers occurs by exchanging the RT loop, in which the n-Src loop acts as a hinge loop, allowing partial exposure of the hydrophobic core of the protein. The open protomer can evolve to form the closed protomer again or, if the concentration is high enough, the hydrophobic residues might avoid solvent exposure by exchanging the RT loop between neighbouring chains. Our results have shown that those variants of the v-Src SH3 in which the dimer is not stabilized are especially prone to aggregation. Whether the lower stability and tendency towards aggregation of full v-Src is related to the lower stability and aggregation behaviour of its SH3 domain requires further characterization.

## 5. Conclusions   

The c-Src kinase is involved in maintaining normal cell homeostasis, and several mechanisms strictly regulate its level of expression and activity. When some of these mechanisms fail, Src is overexpressed or hyperactivated, starting the uncontrolled proliferation of cells that leads to cancer (Sen & Johnson, 2011 ▸). Fifty years ago, Src was the first oncogene to be discovered, in a chicken retrovirus, Rous sarcoma virus (RSV). Since then, the protein codified by this proto-oncogene has been broadly studied to determine the origin of its unregulated behaviour. Nevertheless, to date no structural information has been available. In this work, we have described the first crystallographic structures of the SH3 domain bearing the oncogenic mutations of v-Src. Our results show that a v-Src SH3 variant bearing mutations in the n-Src and distal loops, the v-Src SH3 W95R-I96T variant, shows reduced stability compared with the c-Src SH3 domain but is still able to form intertwined dimers. However, variants bearing mutations in the RT loop cannot form the intertwined dimers. Instead, these proteins are very prone to forming aggregates of high molecular weight in a temperature-dependent manner. Comparing structures of the domain bearing the oncogenic mutations with the inactive conformation of the c-Src tyrosine kinase from chicken reveals the loss of some interactions that might be critical in stabilizing the close-packed inactive state. Additionally, a comparison with structures of the c-Src SH3 domain in complex with PRMs shows that the mutated residues do not affect complex formation. The crystallographic structures in this work might explain the behaviour of the v-Src kinase, which cannot be autoinhibited by binding to the SH2–SH1 linker but still retains its capacity to recognize partner proteins to phosphorylate. Finding the molecular basis of the misbehaviour of the oncogenic proteins is critical for the development of new therapies to fight cancer.

## Supplementary Material

PDB reference: v-Src SH3 domain, Q128R mutant, 7ner

PDB reference: N117D-V124L mutant, 7nes

PDB reference: W95R-I96T mutant, 7net

Supplementary Table S1. DOI: 10.1107/S2059798321004344/gm5080sup1.pdf

## Figures and Tables

**Table 1:** Protein crystallization

Protein	v-Src SH3 Q128R	v-Src SH3 N117D-V124L	v-Src SH3 W95R-I96T
Space group	*P*2_1_	*P*3_2_21	*P*6_5_
Method	Vapour diffusion	Vapour diffusion	Vapour diffusion
Plate type	Sitting drop	Sitting drop	Sitting drop
Temperature (K)	283	288	298
Protein concentration (mg ml^−1^)	5	10	10
Buffer composition of protein solution	10 m*M* Tris buffer pH 8.0	10 m*M* Tris buffer pH 8.0	10 m*M* Tris buffer pH 8.0
Composition of reservoir solution	2.1 *M* ammonium sulfate, 5% PEG 200, 10% glycerol, 40 m*M* lithium chloride, 0.1 *M* acetate pH 5.5	2.8 *M* ammonium sulfate, 0.1 *M* HEPES pH 7.0	2.0 *M* ammonium sulfate, 5% PEG 300, 10% glycerol, 40 m*M* lithium chloride, 0.1 *M* MES pH 6.5
Volume and ratio of the drop	6 µl, 1:1 ratio protein:reservoir solution	6 µl, 1:1 ratio protein:reservoir solution	6 µl, 1:1 ratio protein:reservoir solution
Volume of reservoir (µl)	200	200	200
Observations	1 µl seed solution was added to the crystallization drop	1 µl 0.5 *M* glycine was added to the crystallization drop	

**Table 2:** Data-collection and refinement statistics Values in parentheses are for the highest resolution shell.

	v-Src SH3 Q128R	v-Src SH3 N117D-V124L	v-Src SH3 W95R-I96T
	Monomer	Monomer	Intertwined dimer
PDB entry	7ner	7nes	7net
Beamline	ID30B, ESRF	ID30B, ESRF	XALOC, ALBA
Data-collection temperature (K)	100	100	100
Wavelength (Å)	0.9686	0.9762	0.9791
Resolution range (Å)	19.08–1.55 (1.60–1.55)	18.59–1.35 (1.40–1.35)	19.26–1.50 (1.55–1.50)
Space group	*P*2_1_	*P*3_2_21	*P*6_5_
*a*, *b*, *c* (Å)	22.34, 34.98, 31.14	37.17, 37.17, 65.82	46.65, 46.65, 127.97
α, β, γ (°)	90, 96.40, 90	90, 90, 120	90, 90, 120
Total reflections	18418 (843)	91544 (4717)	83941 (2306)
Unique reflections	6888 (337)	12108 (600)	24714 (1007)
Multiplicity	2.7 (2.5)	7.6 (7.9)	3.4 (2.3)
Completeness (%)	97.9 (94.5)	100 (100)	98.3 (83.3)
Mean *I*/σ(*I*)	6.5 (1.9)	18.1 (2.5)	20.3 (1.9)
Wilson *B* factor (Å^2^)	14.77	15.79	25.26
*R*_merge_	0.049 (0.311)	0.043 (0.707)	0.023 (0.377)
*R*_meas_	0.052 (0.352)	0.049 (0.814)	0.032 (0.522)
*R*_p.i.m._	0.037 (0.249)	0.018 (0.400)	0.016 (0.326)
CC_1/2_	0.998 (0.831)	1 (0.922)	0.999 (0.817)
CC*	1 (0.960)	1 (0.987)	1 (0.954)
Reflections used in refinement	6879 (679)	12056 (1193)	24655 (2190)
Reflections used for *R*_free_	337 (34)	612 (54)	1223 (104)
*R*_work_	0.147 (0.203)	0.158 (0.233)	0.185 (0.241)
*R*_free_	0.162 (0.179)	0.183 (0.234)	0.194 (0.289)
CC(work)	0.976 (0.935)	0.973 (0.950)	0.961 (0.910)
CC(free)	0.972 (0.897)	0.947 (0.934)	0.960 (0.880)
No. of non-H atoms			
Total	544	546	1062
Macromolecules	478	479	952
Ligands	36	0	97
Solvent	48	67	67
No. of protein residues	60	59	114
R.m.s.d., bonds (Å)	0.011	0.010	0.018
R.m.s.d., angles (°)	0.96	1.00	1.60
Ramachandran favoured (%)	96.55	98.21	99.09
Ramachandran allowed (%)	3.45	1.79	0.91
Ramachandran outliers (%)	0.00	0.00	0.00
Rotamer outliers (%)	0.00	1.96	0.00
Clashscore	0.00	0.00	0.52
Average *B* factor (Å^2^)			
Overall	18.69	20.71	38.46
Macromolecules	16.67	19.31	37.56
Ligands	37.77	—	50.64
Solvent	31.73	30.68	43.46

**Table 3:** Apparent p*K*_a_ values for acidic and basic denaturation Values are given with their standard deviation.

	p*K*_a_		No. of ionizable residues					
Protein	Acidic	Basic	Asp	Glu	His	Tyr	Lys	Arg
v-Src SH3 Q128R	4.8 ± 0.1	10.6 ± 0.1	3	4	1	4	2	2
v-Src SH3 W95R-I96T	3.2 ± 0.1	10.4 ± 0.1	3	4	1	4	2	2
v-Src SH3 N117D-V124L	4.9 ± 0.1	10.7 ± 0.1	4	4	1	4	2	1
c-Src SH3 WT†	4.0 ± 0.1	10.8 ± 0.1	4	4	1	4	2	2

† Data from Plaza-Garrido *et al.* (2020 ▸).

**Table 4:** Thermodynamic parameters derived from GdnHCl denaturation

	*m*_1/2_ (*M*)	Δ*G*_w_ (kJ mol^−1^)
v-Src SH3 Q128R	1.74 ± 0.02	9.78 ± 1.04
v-Src SH3 N117D-V124L	2.18 ± 0.02	11.68 ± 1.40
v-Src SH3 W95R-I96T	2.27 ± 0.02	11.43 ± 2.26
c-Src SH3†	2.76 ± 0.03	17.11 ± 2.00

† Data from Plaza-Garrido *et al.* (2020 ▸).

## References

[bb1] Adams, P. D., Afonine, P. V., Bunkóczi, G., Chen, V. B., Davis, I. W., Echols, N., Headd, J. J., Hung, L.-W., Kapral, G. J., Grosse-Kunstleve, R. W., McCoy, A. J., Moriarty, N. W., Oeffner, R., Read, R. J., Richardson, D. C., Richardson, J. S., Terwilliger, T. C. & Zwart, P. H. (2010). *Acta Cryst.* D**66**, 213–221. 10.1107/S0907444909052925 PMC2815670 20124702

[bb2] Afonine, P. V., Grosse-Kunstleve, R. W., Echols, N., Headd, J. J., Moriarty, N. W., Mustyakimov, M., Terwilliger, T. C., Urzhumtsev, A., Zwart, P. H. & Adams, P. D. (2012). *Acta Cryst.* D**68**, 352–367. 10.1107/S0907444912001308 PMC3322595 22505256

[bb3] Arold, S., O’Brien, R., Franken, P., Strub, M.-P., Hoh, F., Dumas, C. & Ladbury, J. E. (1998). *Biochemistry*, **37**, 14683–14691. 10.1021/bi980989q 9778343

[bb4] Bacarizo, J. & Camara-Artigas, A. (2013). *Acta Cryst.* D**69**, 756–766. 10.1107/S0907444913001522 23633584

[bb5] Bacarizo, J., Martínez-Rodríguez, S. & Cámara-Artigas, A. (2015). *J. Struct. Biol.* **189**, 67–72. 10.1016/j.jsb.2014.11.004 25447263

[bb6] Bacarizo, J., Martinez-Rodriguez, S., Martin-Garcia, J. M., Andujar-Sanchez, M., Ortiz-Salmeron, E., Neira, J. L. & Camara-Artigas, A. (2014). *PLoS One*, **9**, e113224. 10.1371/journal.pone.0113224 PMC4260792 25490095

[bb97] Bagnato, G., Leopizzi, M., Urciuoli, E. & Peruzzi, B. (2020). *Int. J. Mol. Sci.* **21**, 2675. 10.3390/ijms21082675 PMC7215861 32290470

[bb7] Baker, D., Riddle, D. S., Grantcharova, V. P., Santiago, J. V., Alm, E. & Ruczinski, I. (1999). *Nat. Struct. Biol.* **6**, 1016–1024. 10.1038/14901 10542092

[bb8] Bergfors, T. (2003). *J. Struct. Biol.* **142**, 66–76. 10.1016/s1047-8477(03)00039-x 12718920

[bb9] Bjorge, J. D., Jakymiw, A. & Fujita, D. J. (2000). *Oncogene*, **19**, 5620–5635. 10.1038/sj.onc.1203923 11114743

[bb98] Brown, M. T. & Cooper, J. A. (1996). *Biochim. Biophys. Acta*, **1287**, 121–149. 10.1016/0304-419x(96)00003-0 8672527

[bb10] Cámara-Artigas, A. (2016). *Arch. Biochem. Biophys.* **602**, 116–126. 10.1016/j.abb.2016.02.024 26924596

[bb11] Cámara-Artigas, A., Martín-García, J. M., Morel, B., Ruiz-Sanz, J. & Luque, I. (2009). *FEBS Lett.* **583**, 749–753. 10.1016/j.febslet.2009.01.036 19185573

[bb12] Chen, V. B., Arendall, W. B., Headd, J. J., Keedy, D. A., Immormino, R. M., Kapral, G. J., Murray, L. W., Richardson, J. S. & Richardson, D. C. (2010). *Acta Cryst.* D**66**, 12–21. 10.1107/S0907444909042073 PMC2803126 20057044

[bb14] Emsley, P. & Cowtan, K. (2004). *Acta Cryst.* D**60**, 2126–2132. 10.1107/S0907444904019158 15572765

[bb15] Emsley, P., Lohkamp, B., Scott, W. G. & Cowtan, K. (2010). *Acta Cryst.* D**66**, 486–501. 10.1107/S0907444910007493 PMC2852313 20383002

[bb88] Engen, J. R., Wales, T. E., Hochrein, J. M., Meyn, M. A. III, Banu Ozkan, S., Bahar, I. & Smithgall, T. E. (2008). *Cell. Mol. Life Sci.* **65**, 3058–3073. 10.1007/s00018-008-8122-2 PMC9357288 18563293

[bb16] Esposito, G., Ricagno, S., Corazza, A., Rennella, E., Gümral, D., Mimmi, M. C., Betto, E., Pucillo, C. E. M., Fogolari, F., Viglino, P., Raimondi, S., Giorgetti, S., Bolognesi, B., Merlini, G., Stoppini, M., Bolognesi, M. & Bellotti, V. (2008). *J. Mol. Biol.* **378**, 887–897. 10.1016/j.jmb.2008.03.002 18395224

[bb17] Evans, P. R. (2011). *Acta Cryst.* D**67**, 282–292. 10.1107/S090744491003982X PMC3069743 21460446

[bb18] Falsone, S. F., Leptihn, S., Osterauer, A., Haslbeck, M. & Buchner, J. (2004). *J. Mol. Biol.* **344**, 281–291. 10.1016/j.jmb.2004.08.091 15504417

[bb93] Gasteiger, E., Hoogland, C., Gattiker, A., Duvaud, S., Wilkins, M. R., Appel, R. D. & Bairoch, A. (2005). The Proteomics Protocols Handbook, edited by J. M. Walker, pp. 571–607. Totowa: Humana Press.

[bb19] Grantcharova, V. P., Riddle, D. S. & Baker, D. (2000). *Proc. Natl Acad. Sci. USA*, **97**, 7084–7089. 10.1073/pnas.97.13.7084 PMC16503 10860975

[bb20] Grantcharova, V. P., Riddle, D. S., Santiago, J. V. & Baker, D. (1998). *Nat. Struct. Mol. Biol.* **5**, 714–720. 10.1038/1412 9699636

[bb21] Hatters, D. M. & Griffin, M. D. (2011). *Methods Mol. Biol.* **752**, 121–136. 10.1007/978-1-60327-223-0_8 21713634

[bb22] Huculeci, R., Garcia-Pino, A., Buts, L., Lenaerts, T. & van Nuland, N. (2015). *Protein Sci.* **24**, 1964–1978. 10.1002/pro.2806 PMC4815226 26384592

[bb23] Joosten, R. P., Long, F., Murshudov, G. N. & Perrakis, A. (2014). *IUCrJ*, **1**, 213–220. 10.1107/S2052252514009324 PMC4107921 25075342

[bb24] Juanhuix, J., Gil-Ortiz, F., Cuní, G., Colldelram, C., Nicolás, J., Lidón, J., Boter, E., Ruget, C., Ferrer, S. & Benach, J. (2014). *J. Synchrotron Rad.* **21**, 679–689. 10.1107/S160057751400825X PMC4073956 24971961

[bb25] Kabsch, W. (1976). *Acta Cryst.* A**32**, 922–923.

[bb26] Kabsch, W. (2010). *Acta Cryst.* D**66**, 125–132. 10.1107/S0907444909047337 PMC2815665 20124692

[bb78] Kato, J. Y., Takeya, T., Grandori, C., Iba, H., Levy, J. B. & Hanafusa, H. (1986). *Mol. Cell. Biol.* **6**, 4155–4160. 10.1128/mcb.6.12.4155-4160.1986 PMC367194 2432397

[bb27] Kilambi, K. P. & Gray, J. J. (2012). *Biophys. J.* **103**, 587–595. 10.1016/j.bpj.2012.06.044 PMC3414879 22947875

[bb77] Klimov, D. K. & Thirumalai, D. (2002). *J. Mol. Biol.* **317**, 721–737. 10.1006/jmbi.2002.5453 11955020

[bb29] Krissinel, E. (2011). *Acta Cryst.* D**67**, 376–385. 10.1107/S0907444911007232 PMC3069753 21460456

[bb87] Levinson, A. D., Oppermann, H., Levintow, L., Varmus, H. E. & Bishop, J. M. (1978). *Cell*, **15**, 561–572. 10.1016/0092-8674(78)90024-7 214242

[bb99] Liebschner, D., Afonine, P. V., Baker, M. L., Bunkóczi, G., Chen, V. B., Croll, T. I., Hintze, B., Hung, L.-W., Jain, S., McCoy, A. J., Moriarty, N. W., Oeffner, R. D., Poon, B. K., Prisant, M. G., Read, R. J., Richardson, J. S., Richardson, D. C., Sammito, M. D., Sobolev, O. V., Stockwell, D. H., Terwilliger, T. C., Urzhumtsev, A. G., Videau, L. L., Williams, C. J. & Adams, P. D. (2019). *Acta Cryst.* D**75**, 861–877. 10.1107/S2059798319011471 PMC6778852 31588918

[bb30] McCarthy, A. A., Barrett, R., Beteva, A., Caserotto, H., Dobias, F., Felisaz, F., Giraud, T., Guijarro, M., Janocha, R., Khadrouche, A., Lentini, M., Leonard, G. A., Lopez Marrero, M., Malbet-Monaco, S., McSweeney, S., Nurizzo, D., Papp, G., Rossi, C., Sinoir, J., Sorez, C., Surr, J., Svensson, O., Zander, U., Cipriani, F., Theveneau, P. & Mueller-Dieckmann, C. (2018). *J. Synchrotron Rad.* **25**, 1249–1260. 10.1107/S1600577518007166 PMC6038607 29979188

[bb31] Miyazaki, K., Senga, T., Matsuda, S., Tanaka, M., Machida, K., Takenouchi, Y., Nimura, Y. & Hamaguchi, M. (1999). *Biochem. Biophys. Res. Commun.* **263**, 759–764. 10.1006/bbrc.1999.1464 10512753

[bb32] Northey, J. G. B., Di Nardo, A. A. & Davidson, A. R. (2002). *Nat. Struct. Biol.* **9**, 126–130. 10.1038/nsb748 11786916

[bb33] Pace, C. N. & Laurents, D. V. (1989). *Biochemistry*, **28**, 2520–2525. 10.1021/bi00432a026 2499351

[bb96] Parsons, S. J. & Parsons, J. T. (2004). *Oncogene*, **23**, 7906–7909. 10.1038/sj.onc.1208160 15489908

[bb34] Pellegrini, E., Piano, D. & Bowler, M. W. (2011). *Acta Cryst.* D**67**, 902–906. 10.1107/S0907444911031210 21931222

[bb35] Plaza-Garrido, M., Salinas-García, M. C., Martínez, J. C. & Cámara-Artigas, A. (2020). *J. Biol. Inorg. Chem.* **25**, 621–634. 10.1007/s00775-020-01785-0 32279137

[bb36] Richter, K., Rufer, A. C., Muller, M., Burger, D., Casagrande, F., Grossenbacher, T., Huber, S., Hug, M. N., Koldewey, P., D’Osualdo, A., Schlatter, D., Stoll, T. & Rudolph, M. G. (2020). *J. Biol. Chem.* **295**, 7849–7864. 10.1074/jbc.RA120.012788 PMC7278359 32317279

[bb37] Riddle, D. S., Grantcharova, V. P., Santiago, J. V., Alm, E., Ruczinski, I. & Baker, D. (1999). *Nat. Struct. Mol. Biol.* **6**, 1016–1024. 10.1038/14901 10542092

[bb38] Roskoski, R. (2004). *Biochem. Biophys. Res. Commun.* **324**, 1155–1164. 10.1016/j.bbrc.2004.09.171 15504335

[bb39] Sen, B. & Johnson, F. M. (2011). *J. Signal. Transduct.* **2011**, 865819. 10.1155/2011/865819 PMC3135246 21776389

[bb76] Sequeira, A., Brás, J. L. A., Fernandes, V. O., Guerreiro, C. I. P. D., Vincentelli, R. & Fontes, C. M. G. A. (2017). *Methods Mol. Biol.* **1620**, 113–128. 10.1007/978-1-4939-7060-5_7 28540703

[bb40] Sicheri, F., Moarefi, I. & Kuriyan, J. (1997). *Nature*, **385**, 602–609. 10.1038/385602a0 9024658

[bb41] Trevino, S. R., Schaefer, S., Scholtz, J. M. & Pace, C. N. (2007). *J. Mol. Biol.* **373**, 211–218. 10.1016/j.jmb.2007.07.061 PMC2084202 17765922

[bb42] Vonrhein, C., Flensburg, C., Keller, P., Sharff, A., Smart, O., Paciorek, W., Womack, T. & Bricogne, G. (2011). *Acta Cryst.* D**67**, 293–302. 10.1107/S0907444911007773 PMC3069744 21460447

[bb43] Willard, L., Ranjan, A., Zhang, H., Monzavi, H., Boyko, R. F., Sykes, B. D. & Wishart, D. S. (2003). *Nucleic Acids Res.* **31**, 3316–3319. 10.1093/nar/gkg565 PMC168972 12824316

[bb44] Williams, J. C., Weijland, A., Gonfloni, S., Thompson, A., Courtneidge, S. A., Superti-Furga, G. & Wierenga, R. K. (1997). *J. Mol. Biol.* **274**, 757–775. 10.1006/jmbi.1997.1426 9405157

[bb45] Winn, M. D., Ballard, C. C., Cowtan, K. D., Dodson, E. J., Emsley, P., Evans, P. R., Keegan, R. M., Krissinel, E. B., Leslie, A. G. W., McCoy, A., McNicholas, S. J., Murshudov, G. N., Pannu, N. S., Potterton, E. A., Powell, H. R., Read, R. J., Vagin, A. & Wilson, K. S. (2011). *Acta Cryst.* D**67**, 235–242. 10.1107/S0907444910045749 PMC3069738 21460441

