# Supplementary material for: The impact of oncogenic mutations of the viral Src kinase on the structure and stability of the SH3 domain
Source: Acta Crystallogr D Struct Biol. 2021 May 19;77(Pt 6):854–66. doi: 10.1107/S2059798321004344 (PMC8171063; doi:10.1107/S2059798321004344)
Supplement: Supplementary file 1 [file d-77-00854-sup1.pdf]

STRUCTURAL
BIOLOGY

Volume 77 (2021)

Supporting information for article:

The impact of oncogenic mutations of the viral Src kinase on the structure and stability of the SH3 domain

M. Carmen Salinas-Garcia, Marina Plaza-Garrido and Ana Camara-Artigas

Table S1 Apparent *pKa* values of the ionisable residues calculated with Rosetta.

Residue	v-Src-SH3 Q128R	v-Src-SH3 N117D-V124L	v-Src-SH3 W95R-I96T	6XVN A/B	6XVO A/B
Tyr90	9.8	9.8	9.8	9.5/9.8	9.8/9.8
Asp91	3.0	3.3	3.1	3.1/3.1	3.2/3.2
Tyr92	10.5	10.2	10.8	10.5/11.1	10.5/10.4
Glu93	4.0	4.5	4.6	3.9/4.1	4.5/4.5
Glu97	4.5	4.4	4.5	4.4/4.3	4.4/4.4
Asp99	1.3	2.8	3.1	2.7/2.7	3.1/2.9
Lys103	11.7	10.5	10.6	10.7/10.7	11.6/11.8
Lys104	10.6	10.6	10.7	10.6/10.6	10.6/10.6
Glu106	4.0	4.3	3.3	4.3/4.3	3.7/4.1
Glu115	4.5	4.4	4.0	4.6/NM	4.5/4.5
Asp117	NP	3.7	NP	3.9/3.7	4.1/4.1
His122	6.7	4.4	6.7	5.4/6.4	6.6/6.4
Tyr131	9.8	9.8	10.4	9.8/9.6	9.7/9.7
Tyr136	9.6	9.8	9.8	9.4/9.9	9.7/9.7

NM-not modelled; NP-not present.
